# Current Advances in Aptasensors for Pesticide Detection

**DOI:** 10.1007/s41061-025-00498-9

**Published:** 2025-03-23

**Authors:** Suthira Pushparajah, Mahnaz Shafiei, Aimin Yu

**Affiliations:** School of Science, Computing, and Engineering Technology, Swinburne University of Technology, Hawthorn, VIC 3122 Australia

**Keywords:** Pesticides detection, Aptasensors, Nanomaterials

## Abstract

The increasing use of pesticides necessitates the development of innovative analytical methods to regulate environmental impacts and ensure food safety. Aptamer-based sensors hold great promise for pesticide detection owing to their superior selectivity, stability, repeatability, and regenerative capabilities. Integrated with nanomaterials, aptasensors have demonstrated enhanced sensitivity for detecting a broad range of pesticides. This study first introduces the aptamer binding mechanism and presents the fundamental concept and justification for selecting aptamer over other biorecognition molecules. It then provides a comprehensive review of recent advancements and applications of various types of aptasensors for targeted pesticide detection, including electrochemical, fluorescent, colorimetric, electrochemiluminescent, and surface-enhanced Raman scattering (SERS) aptasensors. Additionally, it offers a comparative analysis of different aptasensors by evaluating their strengths and limitations. Finally, this review discusses strategies, such as advanced Systemic Evolution of Ligands by Exponential Enrichment (SELEX) technique, self-assembled monolayers (SAMs), and the use of antifouling agents to improve the aptamer’s selectivity, signal-to-noise ratio, and mitigate nonspecific adsorption challenges. These developments are essential for creating highly sensitive and selective aptasensors, facilitating their practical use in environmental monitoring and food safety.

## Introduction

A substantial portion of the global population relies on agriculture for their livelihood. Food security has gained significant attention in many countries owing to the world’s population growth. To address the growing need for food, agricultural methods have adapted to include the widespread utilization of resources, sophisticated technology, and pesticides. Among these methods, using pesticides has been considered a useful and efficient way to reach the intended levels of food production. Pesticides are chemicals applied to manage or eliminate pests by interfering with their physiology or behavior during agricultural production or storage. They can enhance the yield, quality, and productivity of agricultural crops [1]. Nevertheless, the widespread application of pesticides in agriculture in recent decades poses a threat to global food safety and has resulted in increased pollution levels in several ecosystems, such as soil and water. Furthermore, direct and extended contact with pesticides has had detrimental impacts on the health of both humans and animals. The World Health Organization estimates that each year, around two million individuals suffer serious self-poisoning because of contact with pesticides [2]. To safeguard people and the environment against pesticide contamination, it is crucial to develop effective methods for pesticide detection and monitoring.

Conventionally, pesticides are identified by applying chromatographic methods, such as high-performance liquid chromatography (HPLC), gas chromatography/mass spectrometry (GC/MS), and liquid chromatography/mass spectrometry (LC/MS) [3, 4]. For example, GC/MS has been applied to distinguish and identify multi-pesticide traces in food products such as fruits (e.g., apple, grape, and nectarine) and vegetables (e.g., cucumber, spinach, and tomato) [5]. Conversely, LC/MS makes it possible to detect pesticide residues in a rapid and highly accurate manner within a short period of time. This situation persists even if many substances have been examined rarely in relation to food or have proven complicated to analyze using labor-intensive and lengthy approaches [6]. Later, other approaches such as colorimetry [7], surface-enhanced Raman spectroscopy (SERS) [8], capillary electrophoresis [9], and biosensors [10] have also been developed for pesticide identification.

Among these techniques, biosensors have received significant attention owing to their excellent selectivity, simplicity, and suitability for on-site monitoring of traces [11]. Biosensors that integrate with biological recognition molecules, such as enzymes, DNA, or antibodies, can precisely identify their target analytes. For instance, Phongphut et al. have developed an acetylcholinesterase-based biosensor for the successful detection of carbaryl and chlorpyrifos (CPF) [12]. However, enzyme-based sensors suffer from stability issues that may limit their practical use [13, 14]. Immunosensors can conduct sensitive and specific quantitative analyses considerably quicker than traditional immunoassays [15]. For example, an immunosensor for Malathion (MAL) detection has been developed by Kaur et al. using MAL-specific polyclonal antibodies [16]. However, the use of antibodies in biosensor applications is not often applied for pesticide detection because of the challenges and risks associated with antibody preparation for pesticides [17]. To overcome these limitations, aptamers have been developed as alternative biorecognition components to use in the pesticide detection.

## Aptamer Binding Mechanism and Aptasensors

**Fig. 1:**
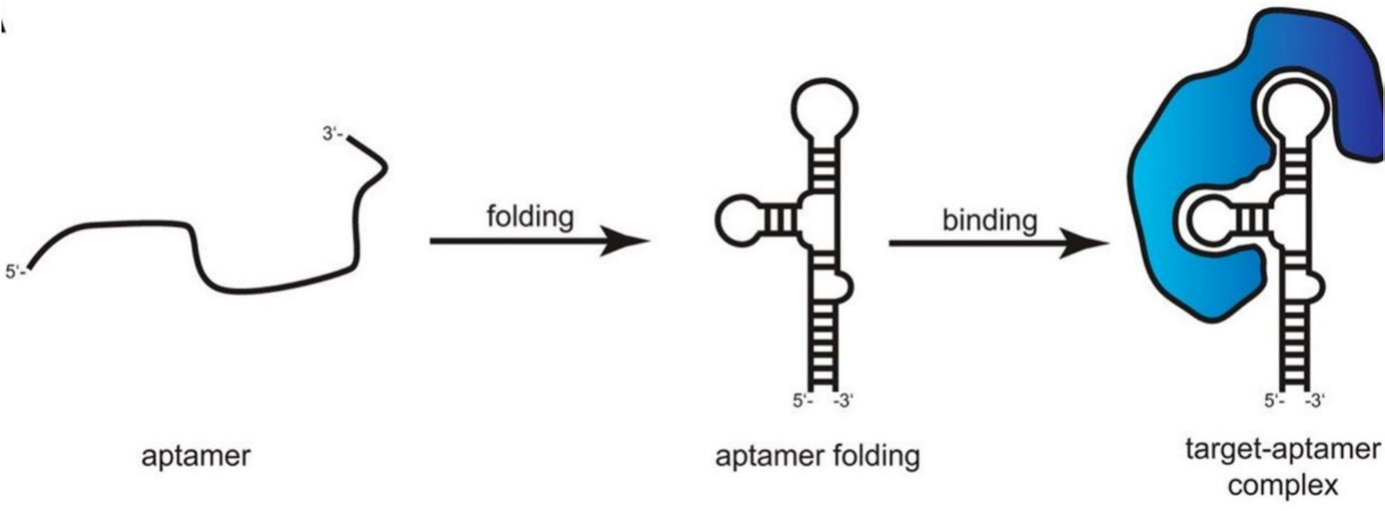
A graphical illustration of the interaction between an aptamer and a target molecule. The aptamer adopts its 3D conformation, enabling it to bind to the intended molecule whenever the binding conditions are altered. The target and aptamer form a stable complex because of this interaction. Adapted with permission [27]. Copyright, 2017 Society for Neuroscience

The word “aptamer” originates in the Latin word “aptus” and the Greek word “meros,” signifying “to fit” and “particle.” Aptamers are short, single-stranded oligonucleotides (DNA, ssDNA, or RNA oligonucleotides) that have been developed by a process known as SELEX that facilitates the identification of molecules with high affinity and specificity for binding. Their length typically varies from 25 to 90 nucleobases and can attach to a diverse range of targets with effective selectivity, allowing them to be beneficial in many applications. Generally, their target molecules vary from small molecules such as toxins [18], drugs, molecular markers, antibiotics [19], hormones [20], ions (heavy metals) [21], peptides, proteins, and small metabolites to complex biological structures such as whole bacterial cells [22], pathogenic bacterial cells [23], and some pesticides [24]. Aptamers must be immobilized on sensor surfaces utilizing methods that maintain their structural integrity and bonding effectiveness to fully exploit their promise in applications, such as biosensing. There are some common methods, such as covalent bonding, physical adsorption, and affinity attachment, to immobilize the aptamers to the electrode surface. Physical adsorption uses noncovalent interactions, while covalent bonding involves forming chemical bonds between aptamer functional groups (e.g., amine, thiol, and biotin) and electrode surface groups (e.g., hydroxyl, amino, and carboxylic). Additionally, affinity molecules, such as streptavidin and biotin improve aptamer binding efficiency. For instance, techniques such as securing biotinylated aptamers onto a sensor surface modified with avidin ensure sufficient surface coverage, stability, and high affinity for binding [25]. As shown in Fig. 1, aptamers have the capacity to fold via the interaction of nucleobases to form a three-dimensional (3D) structure that fits the shape of a target molecule. They use hydrogen bonds, electrostatic interactions, van der Waals forces, aromatic ring stacking, and shape matching to develop an aptamer-target complex [26].

The distinctive folding capability of aptamers enables them to be particularly efficient recognition elements in aptasensors, which provide many benefits. Firstly, aptamers are smaller than antibodies, increasing binding yield or affinity constant with targets, especially in micro- to pico-scale ranges [28]. Increased densities of receptor surface are achievable owing to the compact size, facilitating the binding of many receptors to analytes and minimizing the potential of steric hindrance in sandwich studies. For example, field-effect transistor devices require receptors capable of binding to molecules inside the Debye length (~ 3 nm); however, antibodies measuring roughly 10 nm often surpass this range. Conversely, aptamers fall into the Debye range of 1 to 2 nm [29]. Secondly, aptamers are not harmful to organisms and do not induce immunogenicity since they are manufactured without the use of animals [30]. To eliminate the batch-to-batch variation that are seen in antibody manufacturing, aptamers are subjected to a procedure of high-degree purification. Thirdly, aptamers have stability under various conditions, making them ideal for use in harsh environments, including exposure to organic solvents or elevated salt concentrations without losing their binding affinity. Conversely, antibodies are more susceptible to chemical degradation. They can undergo oxidation, deamidation, and aggregation upon exposure to reactive oxygen species, light, or heat, thereby weakening their effectiveness [31, 32]. Fourthly, aptamers can endure multiple cycles of denaturation and renaturation. Following heat denaturation, aptamers are able to revert back to their original, active configuration [33]. While for antibodies, heat treating can cause permanent unfolding and aggregation and eliminate their biorecognition features [34]. On the basis of its working principle and promising characteristics of aptamer, the following sections comprehensively highlight the recent advancement and application of various aptasensors for pesticide detection.

## Electrochemical Aptasensors

Electrochemical aptasensors recognize specific analytes by producing changes in electrochemical signals upon binding with target molecules, thereby transforming biological interactions into quantifiable electrical signals. By further integrating with nanomaterials, such as carbon nanotubes (CNTs), metal nanoparticles (NPs), and graphene derivatives, the performance of electrochemical aptasensors can be enhanced due to increased effective electrode surface area and efficiency of electron transfer [35, 36].

### Voltammetric and Amperometric Aptasensor for Pesticide Detection

The functioning of voltametric aptasensors operates by varying the applied voltage across the reference and working electrodes, measuring the current response as the voltage changes. On the other hand, amperometric aptasensors work at a constant applied voltage, measuring the current response over time. In both cases, the oxidation or reduction process produces a current signal; the amplitude is generally related to the analyte concentration.

**Fig. 2:**
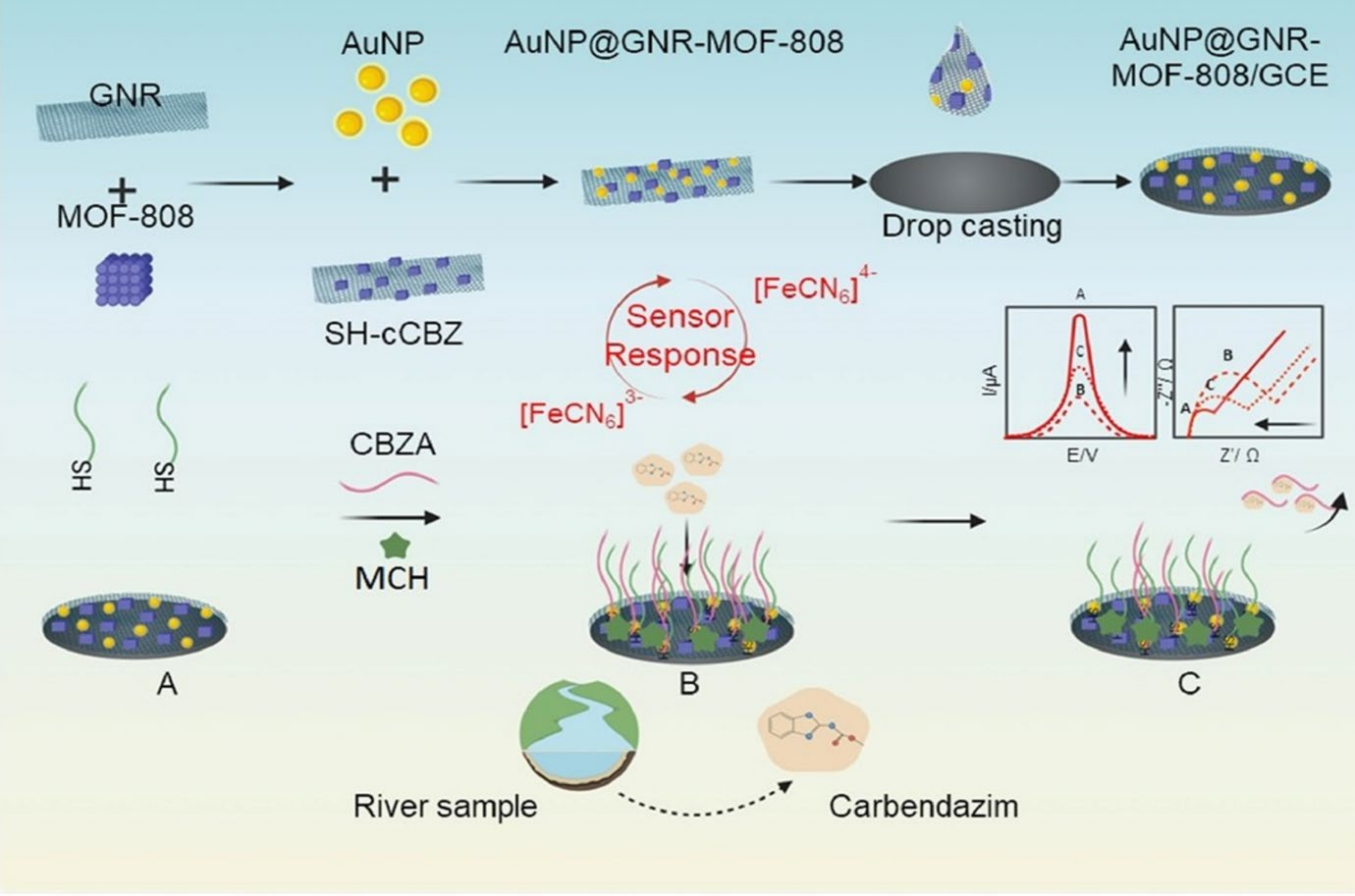
Schematic diagram of CBZ detection through electrochemical aptasensor using MCH-CBZA-SH-cCBZ-AuNP@GNR-MOF-808 deposited on a glassy carbon electrode (GCE). Adapted with permission [38]. Copyright 2022, Elsevier

In a recent example for carbendazim (CBZ) detection, Wang et al. have electrodeposited gold nanoparticles (Au NPs) onto a boron nitride-modified electrode to enhance the conductivity as well as provide a platform for aptamer immobilization via forming Au–S bonds [37]. When CBZ is present and binds to the complementary chain of aptamer, the methylene blue labeled aptamer forms a double-stranded DNA structure, and the oxidation current of methylene blue enhances, proportional to the concentration of CBZ. This aptasensor has shown a linear response to CBZ at concentrations from 520 pM to 0.52 mM. Another study for CBZ detection has been accomplished using a dual-signal-based electrochemical aptasensing platform [38]. Figure 2 illustrates that this sensor employs CBZ aptamer (CBZA) and SH-complementary CBZ aptamer (SH-cCBZ), in conjunction with a zirconium-based metal–organic framework (MOF-808), graphene nanoribbons, and Au NPs. The SH-cCBZ forms a double-stranded DNA structure by binding to CBZA. Upon addition of CBZ, the strong affinity between CBZA and CBZ allows the formation of the CBZ-CBZA complex, which is then eliminated from the electrode surface, resulting in an increased current response of the redox mediator. The innovative dual aptamer design that uses SH-cCBZ and CBZA leads to improved selectivity. The proposed aptasensor exhibits a linear response to CBZ at concentrations from 0.8 fM to 100 pM, with a low LOD of 0.2 fM, which is appropriate for ultra-trace CBZ detection.

Many studies have demonstrated the significance of nanomaterials in sensing performance, playing a key role in improving electrode conductivity, aptamer stability, and signal amplification. For example, two different studies have been reported for the detection of thiamethoxam (TMX). In one work, electrochemical efficiency and electron transport have been enhanced by utilizing composites of porous reduced graphene oxide (PrGO) and Au NPs-doped iron oxide (Au@Fe_3_O_4_) [39]. In another study, Shi et al. also have developed a voltammetric aptasensor for TMX detection utilizing nickel hexacyanoferrate nanoparticles (NiHCF NPs) embedded in cobalt-nitrogen-doped porous carbon (Co–N-C) [40]. The immobilization of NiHCF NPs is shown the enhanced catalytic performance due to Co–N-C, leading to a considerable improvement in the sensing signal. These two TMX studies reported impressive LOD values of 102.7 pM [39] and 12.5 pM [40].

Acetamiprid (ACE) detection has been achieved through a dual signal amplification technique with nanocomposites of Prussian blue (PB) with Au NPs and rGO with silver nanoparticles (Ag NPs) [41]. Modifying rGO and Ag NPs on a GCE increases the sensitivity of the aptasensors by making a high specific surface area for immobilizing substances, thereby enhancing the electrical signal generation. This dual signal amplification system integrates PB and Au NPs, which improves the catalytic efficiency of the sensor, facilitating more effective redox reactions. This aptasensor records a LOD as 0.3 pM. In a similar approach, a DNA amplification platform with mesoporous dendritic fibrous nanosilica (NH_2_-DFNS) has been developed to determine CPF [42]. A nano-Au-coated electrode is functionalized with NH_2_-DFNS using aptamers that selectively recognized the complementary DNA. A key feature of the NH_2_-DFNS platform is its capability to increase DNA loading and use of molybdate-phosphate as signal-enhancing compound. This approach has strengthened the sensor’s conductivity and stability while also improving aptamer-mediated chemical functionalization, enabling the detection of CPF in a minimal LOD of 0.43 fM. Another further instance of composite signal amplification is the development of a MAL voltammetric aptasensor by Ma et al. which, combines black phosphorous (hydroxylated) (hBP), poly-l-lysine (PLL), and Au NPs [43]. The integration of hBP nanosheets with PLL and Au NPs offers a resilient support framework for aptamer immobilization. This composite improves the stability, conductivity, and binding strength of the aptasensor. This aptasensor exhibits a very low LOD of 2.805 fM, being among the lowest recorded for MAL detection.

**Fig. 3:**
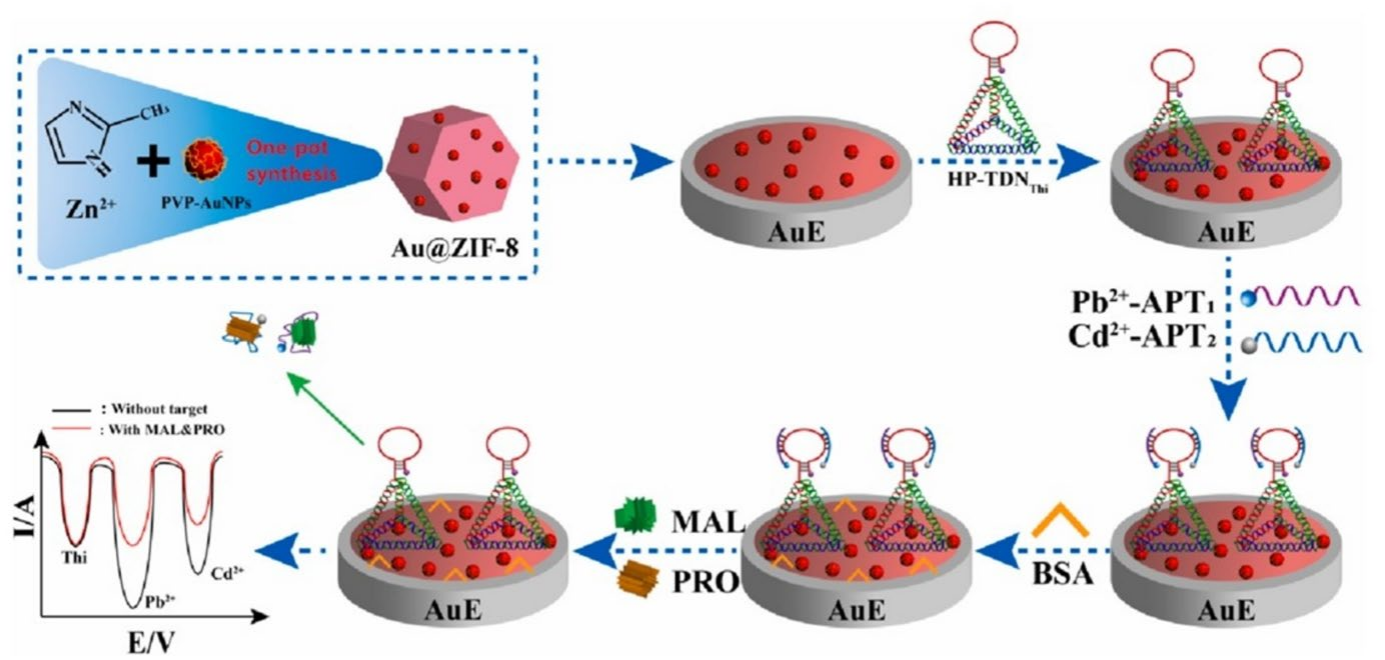
Schematic illustration of MAL and PRO detection through electrochemical aptasensor. Adapted with permission [44]. Copyright 2023, Elsevier

Recent investigations in voltammetric aptasensors discuss the important role of labeling agents, aptamer configurations, and the combination of nanomaterials and labeling agents in enhancing sensitivity and selectivity, respectively. These approaches allow the identification of many pesticides at very low concentrations, even within complicated sample matrices. Labeling agents such as thionine (Thi) and ferrocene (Fc) and different aptamer shapes, such as hairpin structures, have been applied to enhance the signal response, stability, and selectivity, respectively. For instance, as shown in Fig. 3, Li et al. have developed a voltammetric aptasensor for detecting MAL and profenofos (PRO) that uses Thi for hairpin structured aptamer labeling. The application of a hairpin-structured DNA nanostructure framework (HP-TDN) with metal ions as signal tracers is an innovation to improve the stability and efficiency of the sensor [44]. Thi-tagged HP-TDN facilitated the establishment of Pb^2+^-tagged MAL APT1 and Cd^2+^-tagged PRO APT2 via specific binding sites. The detachment of Cd^2+^-PRO APT2 and Pb^2+^-MAL APT1 from HP-TDNThi’s complementary hairpin results in decreased oxidation currents for Cd^2+^ and Pb^2+^ in the presence of target pesticides. The oxidation current of Thi remains unchanged. This is a result of the unique and independent functions of the aptamers for MAL and PRO. The design of the sensor ensures that the variations in oxidation current for Pb^2+^ and Cd^2+^ are monitored separately, allowing for a precise distinction between the two pesticides. This dual-metal-ion approach ensures measurements of MAL and PRO with LODs of 13 pM and 35.6 pM, respectively. The amounts of MAL and PRO are calculated by measuring the ratios of oxidation current of Pb^2+^/Thi and Cd^2+^/Thi. The integration of Au NPs into the ZIF-8 (zeolitic imidazolate framework) nanocomposites improves the signal response and HP-TDN capture.

Similarly, Yu et al. have labeled the 3′-end of the aptamer with a Fc component, which is then immobilized onto an Au electrode surface to detect isocarbophos (ISO) [45]. The inclusion of an azobenzene group at the 5′ end facilitates photo-responsive regulation, providing a distinctive method for adjusting the sensor’s performance. These groups experience a structural transformation from trans to cis upon exposure to UV light, thereby influencing the aptamer’s hairpin configuration and increasing the sensor’s selectivity to ISO. This approach states that an increase in ISO concentration results in an increase in the aptasensor’s current response. This aptasensor shows a broad linear detection range of 10 pM to 10 µM and a low LOD of 3 pM. The integration of Fc components for aptamer immobilization and azobenzene for structural alterations highlights a multimodal design approach, possibly encouraging more biosensor advancements. However, the need for UV light to trigger structural changes in azobenzene may limit the sensor’s practicality, particularly in portable or on-site environments where UV sources may be limited. In another approach, a hairpin-structured aptamer has been used as the signalling donor by Fu et al. to enhance the selectivity of organophosphorous pesticides (OPs) [46]. In this design, the 5′ end of the aptamer has been modified with amino groups, while the 3′ end has been labeled with the redox probe Fc. The strategic positioning of Fc at the 3′ terminus allows accurate monitoring of the conformational change, which closely corresponds with the presence of OPs. Aptamer has immobilized to the GO-chitosan (CS) nanocomposite through an amide bond. CS acting as a stabilizing agent, efficiently prevents the aggregation of carbon-based materials, such as rGO, guaranteeing homogeneous material distribution and enhancing the sensor performance. When an OP has added, the OP has bound with aptamer, causing the hairpin configuration to open. As a result, the Fc moves away from the electrode’s surface, leading to a decrease in the Fc’s redox signal. For this work, the existence of additional electroactive species in intricate matrices may disrupt the redox signal and influence the accuracy of the detection.

The combination of  Thi and Fc with nanoparticles further demonstrates the improved efficiency of voltammetric aptasensors. For instance, an ACE voltammetric aptasensor has been developed with the use of a nanocomposite of hBP, Thi, PLL, and Au NPs [47]. The sensor’s reliability and durability are confirmed by the synergistic effect of Thi (capping agent) and PLL (adhesion agent), which is necessary for practical applications. Although hBP is prone to environmental deterioration, its unique chemical and physical properties provide an ideal material for sensor preparation. This sensor has a broad linear response range from 10 fM to 1 µM for ACE detection. This extended measurement range increases their practical applications in environmental monitoring and food safety. The LOD for this aptasensor is very low, LOD 9.407 fM, indicating their great sensitivity. The primary challenge is that external conditions, such as temperature, time, and humidity may affect these sensors’ efficiency, requiring close observation throughout measurement. In another study, CPF has identified using a combination of Fc-dispersed MWCNTs and mesoporous carbon functionalized by CS [48]. CS evenly distributes Fc and carbon-based materials throughout the composite matrix. CS and carbon-based materials react chemically to improve compatibility and dispersion while CS provides a stable matrix for dispersed particles, retaining their functionality. While these above investigations demonstrate the adaptability and efficiency of electrochemical aptasensors in detecting a broad spectrum of pesticides, Table 1 summarizes recent studies on pesticide detection utilizing electrochemical aptasensors on the basis of voltammetry and amperometry.

**Table 1:** Electrochemical aptasensors for pesticide detection on the basis of voltammetry and amperometry

Materials	Pesticide	Sample	Linear range	LOD	References
Aptamer-BN-Au NPs/GCE	CBZ	Cucumber, tap water, and kiwi	520 pM–0.52 mM	99 pM	[37]
Aptamer-GNR@AuNP-CNHs-MOF/GCE	CBZ	Tap water and river water	0.8 fM–100 pM	0.2 fM	[38]
Aptamer-CNT-COOH/SPCE	CBZ	Tomatoes	1–50 nM	4.35 nM	[10]
Aptamer-PrGO-Au@Fe_3_O_4_/GCE	TMX	Green leafy vegetables	343 pM–3.43 µM	102.7 pM	[39]
Aptamer-Au NPs-NiHCFNPs-Co–N-C/GCE	TMX	Environmental water and potato	34.3 pM–343 nM	12.5 pM	[40]
Aptamer-Pb-Au NPs-rGO-AgNPs/GCE	ACE	Vegetables	1 pM–1 µM	0.3 pM	[41]
Aptamer-hBP-Thi-PLL-Au NPs/GCE	ACE	Tea	10 fM–1 µM	9.407 fM	[47]
Aptamer-PAMAM-PL-PEDOT/GCE	ACE	Vegetables	0.4 pM–44.84 nM	53 fM	[49]
Aptamer-3D-CS-rGO/GCE	ACE	Tea	0.1 pM–0.1 µM	71.2 fM	[50]
Aptamer-[P(AMT)]-Au NPs/SPE	ACE	Tomato, cucumber, and wastewater	5–500 pM	1.5 pM	[51]
Aptamer-Fc@MWCNTs- OMC-CS/GCE	CPF	Vegetables and fruits	2.85 nM–0.285 mM	94 pM	[48]
Aptamer-Au NPs-Mo_2_C-Mo_2_N/GCE	CPF	Apple and pakchoi	0–1.139 µM	0.1026 nM	[52]
DFNS-NH_2_-Au NPs@cDNA-Aptamer-Au/GCE	CPF	Apple and tomato	1 fM–1 nM	0.43 fM	[42]
Thiolated aptamer-Au NPs/SPGE	DZN	Plasma of male wistar rat	0.1–1000 nM	0.0169 nM	[53]
Aptamer-VS_2_QDs-GNP-CMWCNTs/GCE	DZN	River water, soil, apple, and lettuce	50 fM–10 nM	11 fM	[54]
Aptamer-hBP-PLL-Au NPs/GCE	MAL	Lake water, green grocery, cabbage, and soil	0.1 pM–1 µM	2.805 fM	[43]
CP-MOF-Fc-aptamer-Au NPs/GCE	MAL	Cucumber and long bean	75.7 pM–2.57 nM	52 pM	[55]
Aptamer-Au@ZIF-8/AuE	MAL PRO	Lettuce, spinach, and Chinese cabbage	15.1 pM–151 nM 134 pM–134 nM	13 pM 35.6 pM	[44]
Aptamer-Au nanoshell-MWCNT-Gr/SPCE	PRO	Spinach, lettuce, and cabbage	268 pM–268 µM	139 pM	[56]
Aptamer-Fc/AuE	ISO	Tomato	10 pM–10 µM	3 pM	[45]
Aptamer-Fc-GO-chitosan/SPCE	PRO PHR ISO OMT	Rape, cabbage, spinach, and baby cabbage	0.01–1000 nM 0.1–800 nM 0.01–1000 nM 0.1–100 nM	0.01 nM 0.1 nM 0.01 nM 0.1 nM	[46] [46] [46] [46]

*BN*-*Au NPs/GCE* boron nitride-gold nanoparticles/glassy carbon electrode, *GNR-CNHs/MOF* graphene nanoribbons-carbon nanohorns/Zr-based metal–organic framework, *CNT-COOH/SPCE* oxidized carbon nanotubes/screen printed carbon electrode, *PrGO-Fe*_*3*_*O*_*4*_ porous reduced graphene oxide-iron (II-III) oxide, *NiHCF NPs-Co–N-C* nickel hexacyano ferrate nanoparticles-cobalt-nitrogen doped porous carbon, *Pb-rGO-Ag NPs* prussian blue-reduced graphene oxide-silver nanoparticles, *HBP-Thi-PLL* hydroxylated black phosphorus-thionine-poly-L-lysine, *PAMAM-PL-PEDOT* poly(amidoamine)-phaseoloidin-doped poly(3,4-ethyloxythiopene), *3D-CS* three-dimensional chitosan, *P(AMT)/SPE* poly-5-amino-2-mercapto-1,3,4-thiadiazole/screen printed electrode, *Fc@MWCNTs-OMC-CS* ferrocene hybrid chitosan dispersed multiwalled carbon nanotubes-mesoporous carbon functionalized by chitosan, *Mo*_*2*_*C-Mo*_*2*_*N* molybdenum carbide-molybdenum nitride, *DFNS-NH*_*2*_*-cDNA* amino functionalized mesoporous dendritic fibrous nanosilica-complementary DNA, *SPGE*, screen printed gold electrode, *VS*_*2*_*QDs-GNP-CMWCNTs* vanadium disulfide quantum dots-graphene nanoplates-carboxylated multiwalled carbon nanotubes, *CP-Fc* complementary probe-ferrocene, *ZIF/AuE* zeolitic imidazole framework/gold electrode, *Gr* graphitized, *GO* graphene oxide, *CBZ* carbendazim, *TMX* thiamethoxam, *ACE* acetamiprid, *CPF* chlorpyrifos, *DZN* diazinon, *MAL* malathion, *PRO* profenofos, *ISO* isocarbophos, *PHR* phorate, *OMT* omethoate

### Electrochemical Aptasensors Based on Impedance Spectroscopy

**Fig. 4:**
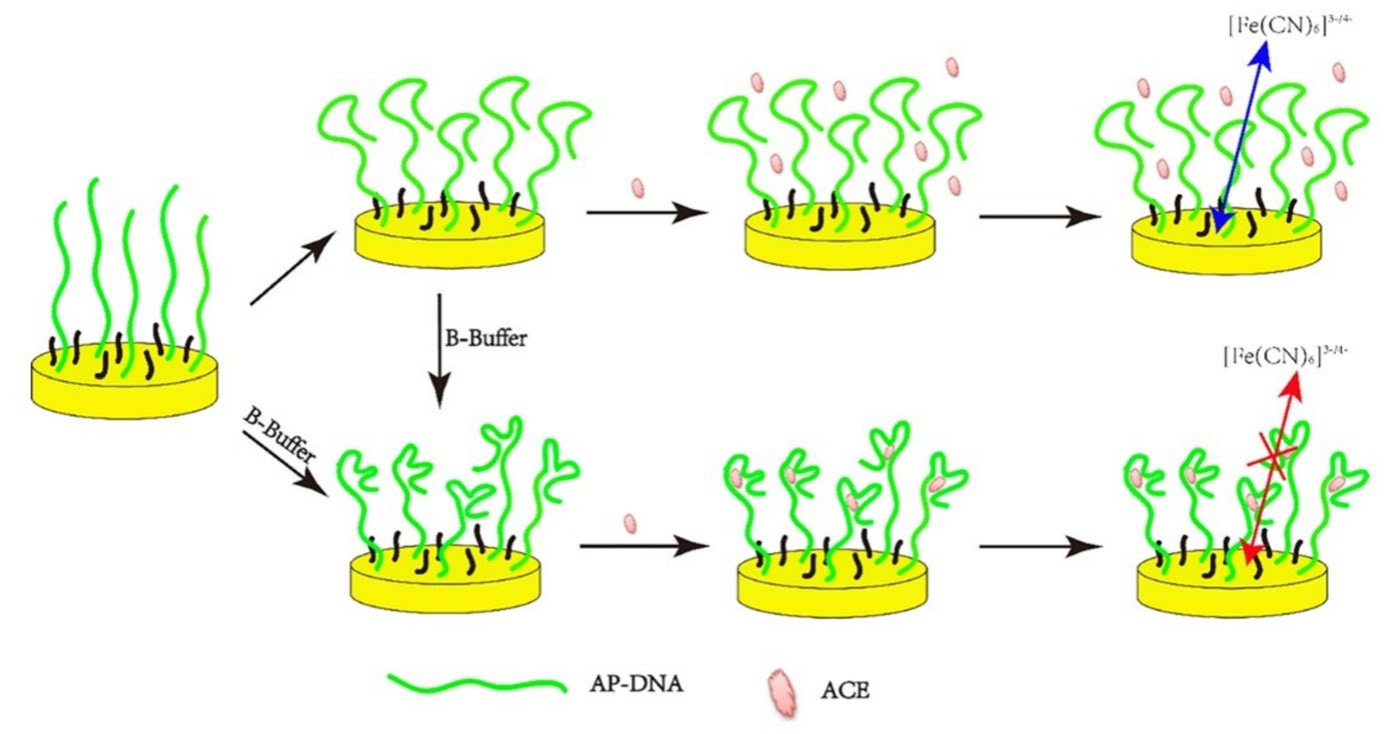
Schematic illustration for an EIS-based aptasensor for ACE detection. Adapted with permission [57]. Copyright 2020, PLOS

The electron transfer in redox reactions at the electrode surface can be influenced by molecule attachment, which results in changes in electrode resistance or impedance. This phenomenon can be monitored through the electrochemical impedance spectroscopy (EIS) technique, which is particularly used for analyzing charge transfer resistance (RCT). In impedance-based aptasensor, the binding of nonconductive target molecules to aptamer-modified electrode surfaces can hinder or diminish the electron transfer of redox probes, resulting in an increase in RCT. This concept has been used for the development of pesticides-specific aptasensors. For instance, Zhen et al. have observed impedance change of Ap-DNA (1) immobilized on an Au electrode for ACE detection [57]. As shown in Fig. 4, single-strand AP-DNA (1) originally exhibits a weakly folded hairpin-like secondary structure. After ACE binds to the stem-loop structure of the aptamer to form the ACE-AP-DNA (1) complex, the electrode’s RCT increases. There is a direct relationship between ∆RCT and the quantity of bound ACE. The aptasensor shows a linear response to ACE at concentrations from 5 nM to 200 mM. The LOD for this proposed aptasensor is 1 nM.

Similarly, another impedimetric aptasensor has been developed by Eissa and Zourob et al. to detect CBZ using a thiol-modified aptamer [58]. The CBZ-specific aptamer experiences a conformational change when CBZ binds, which hinders the redox probe from reaching the electrode. The aptasensor response is determined by monitoring the rise in the RCT of the redox probe using Faradaic EIS. This Faradaic EIS method enhances the sensitivity and accuracy of label-free detection. Moreover, it simplifies assay and reduces any interference from labeling agents. The sensor’s capacity to detect trace amounts of CBZ is shown by the measured LOD of 42.9 pM and a wider linear range of 5.23 pM to 52.3 nM.

To further enhance the efficiency of the impedimetric aptasensor, many studies apply different forms of nanostructures, including graphene derivatives and metal NPs, such as Au and platinum NPs. These nanomaterials contribute to achieving reduced LOD in the fM and pM ranges, therefore enhancing the overall efficiency for precise pesticide detection. The following two examples illustrate the unique functions of Au NPs and Pt NPs in improving sensor efficiency. For example, Roushani et al. have modified GCE with Au nanorods and aptamer-imprinted polymers for CPF detection, resulting in double-specific recognition [59]. The integration of aptamer-imprinted polymers with Au nanorods offers synergistic recognition mechanisms, enhancing higher selectivity with a lower LOD of 0.35 fM. In contrast, Madianos et al. have used Pt NPs microwires modified with aptamers on the interdigitated electrodes (IDEs) to identify atrazine (ATZ) and ACE [60]. The integration of sputtering and e-beam lithography for Pt NPs deposition further provides precise control over the electrode design, hence improving the sensor’s efficiency. This technique appears to be a reliable and label-free method while minimizing operational complication. The aptasensor can detect both ACE and ATZ. The LODs for the proposed aptasensor were 1 pM and 10 pM for ACE and ATZ, respectively.

In addition to metal NPs, different graphene derivatives combined with other nanocomposites have been applied in many impedances-based aptasensors. Three examples describing the impact of different graphene derivatives on sensor performance. For example, Fan et al. have developed an electrochemical aptasensor for ATZ detection using electrochemically rGO (ErGO) and NiHCF NPs [61]. Au NPs have been electrodeposited onto the electrode, which serves as a platform for aptamer immobilization. NiHCF NPs act as a signaling probe while ErGO enhances the electron transfer to improve the overall performance of the sensor. The development of the ATZ-aptamer complex on the sensor surface increases the impedance due to the enhanced steric hindrance of electron transfer from the redox probe, enabling the quantitative identification of ATZ. This sensor records 0.1 pM of LOD, which is very low, enabling this aptasensor to measure ATZ at trace levels. Similarly, Jiao et al. have applied a different graphene derivative, such as graphene oxide (GO)-functionalized CS and carbon black (CB), to design a sensitive impedance-based aptasensor for CPF detection [62]. This combination facilitates the strong attachment of the aptamer onto the electrode surface. The LOD for this suggested aptasensor is 94.1 pM. In another approach, Khosropour et al. have used graphene nanoplatelets-carboxylated (GNPs) combined with c-MWCNTs and doped with vanadium disulfide quantum dots (VS_2_QDs) for the detection of diazinon (DZA) [54]. The DZN-specific aptamer has attached onto the VS_2_QDs-GNP-CMWCNTs-modified electrode via electrostatic interaction. [Fe(CN)_6_]^3−/4−^ serves as a redox indicator for EIS change. The selective adsorption of DZN on the aptamer-modified electrode causes a change in the RCT. The combined effect of VS_2_QDs, GNPs, and CMWCNTs maximises their unique and synergistic properties for signal amplification and more accurate detection. Which further enables a lowered LOD as 2 fM and an expanded linear range from 10 fM to 10 nM. These examples illustrate the distinct characteristics and adaptability of graphene derivatives with other nanocomposites to improve the performance of the sensor for pesticide detection. Table 2 lists some recent studies on pesticide detection utilizing electrochemical aptasensors with impedance changes.

**Table 2:** Electrochemical aptasensors based on impedance changes for pesticide detection

Material used	Pesticide	Sample	Linear range	LOD	References
Aptamer-AuE	ACE	Orange juice	5 nM–200 mM	1 nM	[57]
Aptamer-HPG/AuE	ACE	Apple, pear, orange, cucumber, tomato, and pakchoi	0.5–300 nM	0.34 nM	[63]
Aptamer-GOPTS-PtNPs microwires/Au IDEs	ACE	Tap and bottled mineral water	10 pM–100 nM	1 pM	[60]
	ATZ		100 pM–1 μM	10 pM	[60]
Aptamer-NiHCF NPs-ERGO/GCE	ATZ	Lake and river water	0.25–250 pM	0.1 pM	[61]
Aptamer/AuE	CBZ	Soya milk, mango juice, tomato, and plum fruit	5.23 pM–52.3 nM	42.9 pM	[58]
Aptamer-GNR@AuNP-CNHs-MOF/GCE	CBZ	Tap water and river water	1 fM–100 pM	0.4 fM	[38]
Aptamer-Au NPs-1-AP-CNHs/GCE	CBZ	Lettuce and orange	5.23 pM–5.23 nM	2.62 pM	[64]
Aptamer-CB-CS-GO@Fe_3_O_4_/GCE	CPF	Cabbage, pakchoi, lettuce, and leek	0.285 nM–0.285 mM	94.1 pM	[62]
MIP-aptamer-AuNRs/GCE	CPF	Apple and lettuce	1 fM–0.4 pM	0.35 fM	[59]
Aptamer-VS_2_QDs-GNP-CMWCNTs/GCE	DZN	River water, soil, apple, and lettuce	10 fM–10 nM	2 fM	[54]

*AuE* gold electrode, *HPG* highly porous gold, *GOPTS-PtNPs/IDEs* 3-glycidyloxypropyl triethoxysilane platinum nanoparticles/interdigitated electrodes, *NiHCF NPs-ERGO/GCE* nickel hexacyanoferrate nanoparticles electrochemically reduced graphene oxide/glassy carbon electrode, *GNR@Au NPs-CNHs-MOF* graphene nanoribbons@gold nanoparticles-carbon nanohorns/Zr-based metal–organic framework, *1-AP* 1-aminopyrene, *CB-CS/GO@Fe*_*3*_*O*_*4*_ carbon black-chitosan-graphene oxide@iron oxide, *MIP-AuNRs* molecularly imprinted polymer gold nanorods, *VS*_*2*_*QDs-GNP-CMWCNTs* vanadium disulfide quantum dots-graphene nanoplates-carboxylated multiwalled carbon nanotubes, *ACE* acetamiprid, *ATZ* atrazine, *CBZ* carbendazim, *CPF* chlorpyrifos, *DZN* diazinon

## Optical Aptasensors

The aim of optical aptasensors is to precisely measure the light emitted or absorbed because of a specific biological interaction. These sensors use variations in optical characteristics to identify and quantify the presence of certain substances, such as pesticides. Optical detection techniques include colorimetry, which measures color changes; luminescence, which detects light emission through chemical reactions (chemiluminescence) or electrochemical processes (electrochemiluminescence); and fluorescence, where the sensor detects light emitted by fluorescent substances.

### Colorimetric Aptasensor

Colorimetric aptasensors have been widely used for the detection of pesticides in food and the environment owing to their easy preparation, cost-effectiveness, and the ability to observe results without specialized equipment [65]. Au NPs and Ag NPs are often used as probes in colorimetric sensing because of their size-dependent surface plasmon resonance (SPR) properties, which help to generate colorimetric signals in detection assays [66]. When compared with other metal NPs, AuNPs have drawn greater interest because of their durability and the strong impact of interparticle spacing on their optical features [67]. Two examples of colorimetric aptasensors for pesticide detection based on Au NPs and Ag NPs are discussed below to demonstrate these features.

**Fig. 5:**
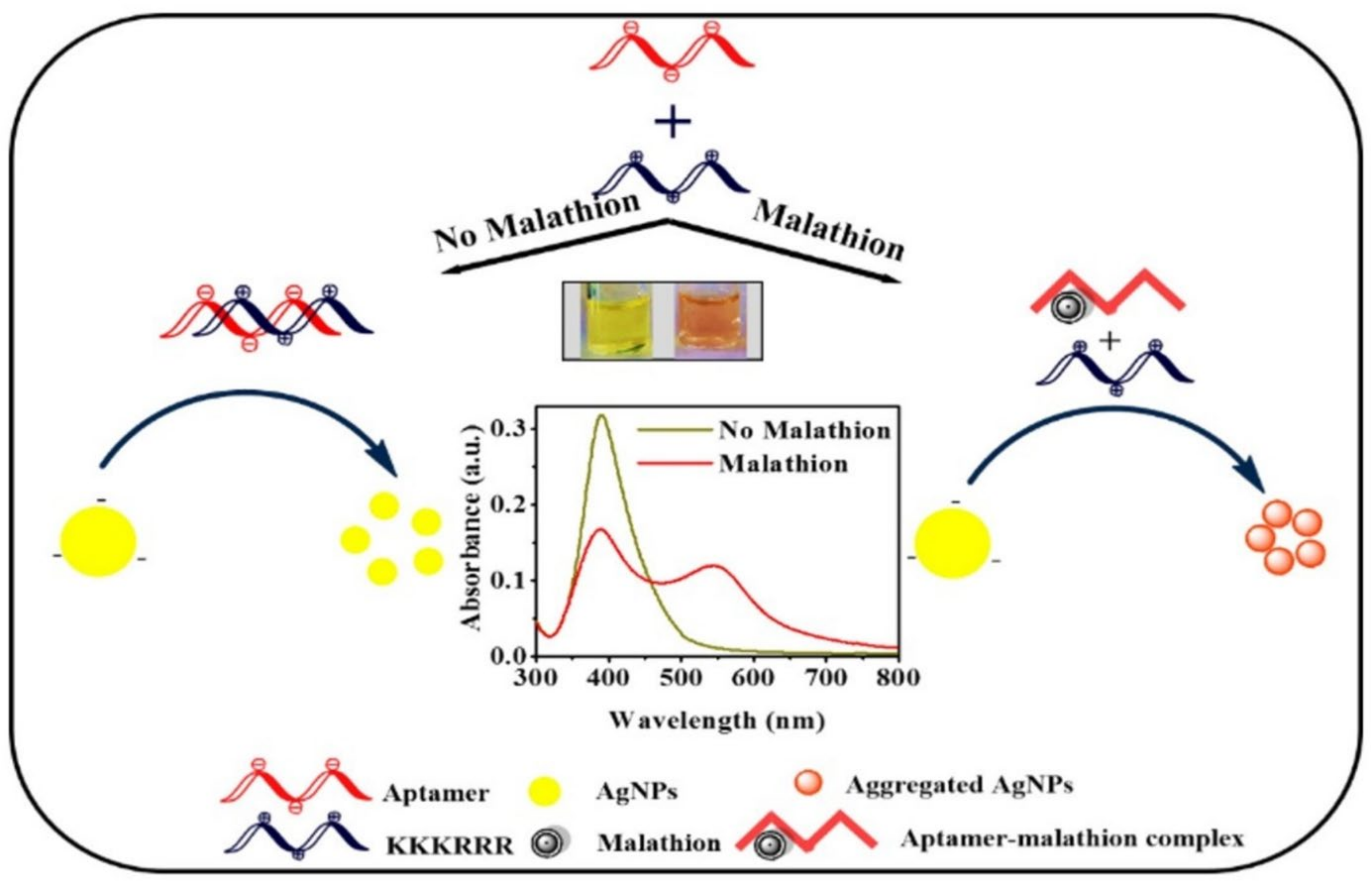
Schematic illustration of colorimetric base aptasensor for MAL detection. Adapted with permission [69]. Copyright 2018, Elsevier

For instance, Wang et al. have developed a colorimetric CBZ aptasensor based on poly-diallyldimethylammonium chloride (PDDA)-induced Au NPs aggregation [68]. In the absence of CBZ, negatively charged Au NPs remain dispersed throughout the solution, whereas the CBZ aptamer forms a complex with the cationic polymer PDDA by electrostatic interaction. The addition of CBZ into the system to form the CBZ-aptamer complex structure would release PDDA, which would then interact with Au NPs, resulting in the aggregation of Au NPs. The PDDA-induced Au NP aggregation causes the increase of particle size, resulting in the SPR peak moving to a longer wavelength. This leads to the solution color change from red to blue, which is made achievable by the aptamer-CBZ interaction. This colorimetric aptasensor shows a linear response to CBZ within a concentration range from 2.2 to 500 nM. The LOD for this proposed aptasensor is 2.2 nM. This method combines molecular recognition with nanotechnology. The colorimetric change enables visual detection without advanced instrumentation, which benefits on-site applications. While Au NPs are commonly used, Ag NPs are also applied as an alternative in colorimetric assays. For instance, as shown in Fig. 5, Bala et al. have developed an aptasensor using Ag NPs as a nanoprobe, a hexapeptide KKKRRR, and a MAL-specific aptamer [69]. In the absence of MAL, the positively charged peptide and negatively charged aptamer form an aptamer-peptide complex, and Ag NPs are evenly distributed in solution. The unique conformational shift from a random coil to a rigid shape is brought about by the presence of MAL, which causes the MAL to bind specifically with the aptamer and further ensures a specific and measurable response. The interaction between negatively charged Ag NPs and free peptide causes the aggregation of Ag NPs because of the MAL addition. The increase in particle size of Ag NPs alters the properties of SPR by shifting the SPR peak to a longer wavelength. The resulting solution color changes from yellow to orange, offering a simple and visible detection technique. This aptasensor improved the performance in terms of sensitivity, reaching a 0.5 pM of LOD, making it appropriate for detecting MAL even at trace quantities. It is noted that the aforementioned studies may experience an aggregation process that may be susceptible to differences in experimental factors, such as ionic strength, pH, and temperature, which might affect reproducibility and stability. Besides the above examples, Table 3 provides a summary of recent colorimetric aptasensors for pesticide detection, highlighting their broad range of applications in the food safety and environmental field.

**Table 3:** Colorimetric aptasensors used for pesticide detection

Pesticide	Sample	Linear range	LOD	References
Carbendazim	Water	2.2–500 nM	2.2 nM	[68]
Carbendazim	Soybean	8.16 µM–0.131 mM	8.16 µM	[70]
Imidacloprid		12.2–97.8 µM	12.2 µM	[70]
Malathion	Apple, tap water, and lake water	0.01 nM–0.75 nM	0.5 pM	[69]
Acetamiprid	Chinese cabbage and cucumber	2.3–27 µM	1.75 µM	[71]
Acetamiprid	Cabbage, cucumber, and river water	0–140 µM	1.74 µM	[72]
Chlorpyrifos	Tap water, cucumber, and cabbage	200–5000 nM	14.46 nM	[73]
Paraquat	Agriculture soil, tap water, and pond water	5–50 nM	2.76 nM	[74]

### Fluorescence Resonance Energy Transfer (FRET) Based Aptasensors

Fluorescence resonance energy transfer (FRET) involves exciting a donor fluorophore with light that can transfer energy to a neighboring acceptor molecule, causing a decrease in both the lifespan and intensity of fluorescence of the donor molecule [75]. The changes in fluorescence intensity depend on the binding of the target, which enables precise quantification of pesticide concentrations. The accuracy of this measurement can be improved through including nanomaterials, such as carbon dots, QDs, up-conversion NPs (UC NPs), GO, and Au NPs [76]. To demonstrate this FRET concept, two instances of nanomaterial-based fluorescent aptasensors for pesticide detection are discussed below.

**Fig. 6:**
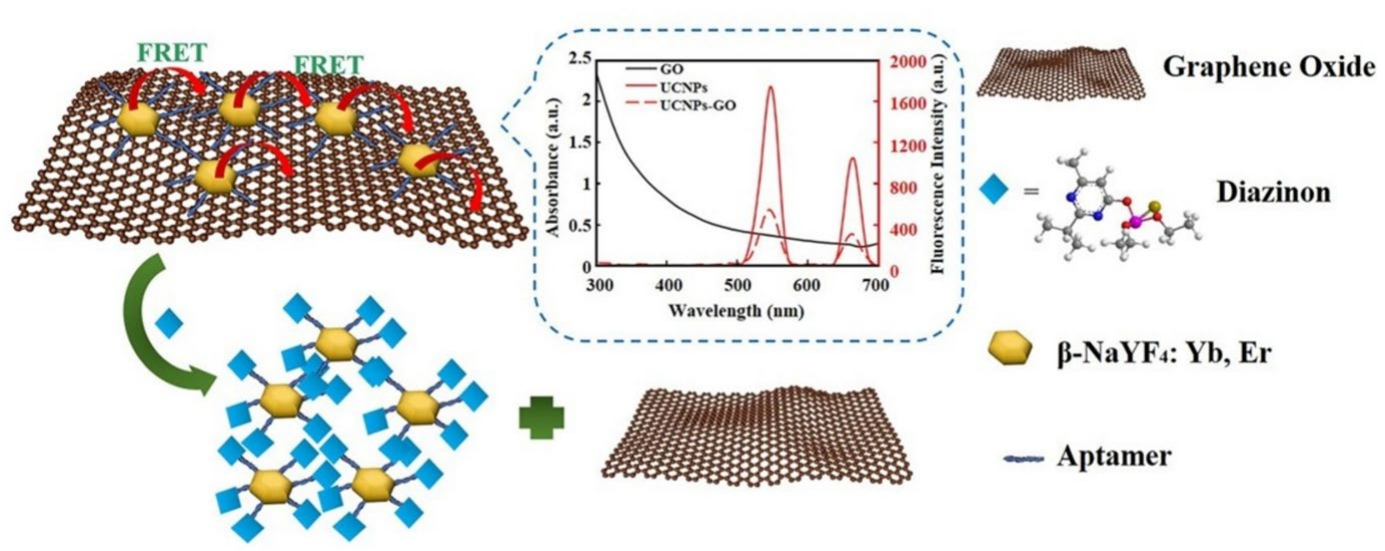
Schematic diagram of UC NPs-based FRET for DZN detection. Adapted with permission [78]. Copyright 2020, Elsevier

For instance, a GO-based FRET sensor has developed to detect edifenphos [77]. In this study, the recognition component consisted of aptamers, zinc sulfide QDs, and GO, which serve as the donor and acceptor, respectively. The adsorption of the QD-aptamer onto GO reduces the intensity of the QDs’ fluorescence emission. The QD-aptamer is freed from the GO sheets when Edifenphos is present, which allows the fluorescence intensity to be fully recovered. This reversible fluorescence recovery enables real-time monitoring, with a LOD of 0.419 nM. Another study has applied the UC NPs for the FRET technique for DZN detection [78]. UC NPs provide quick detection by converting near-infrared light into visible light, hence enhancing the fluorescence detection. The fluorescence quenching and amplification mechanism resulting from the FRET interaction between UC NPs and GO simplifies the procedure and eliminates the need for complex equipment. The design of UC NPs modified with aptamers customizes this system for the detection of targets, hence improving both selectivity and sensitivity. The application of π–π interactions for conjugating aptamer-UC NPs with GO is an important feature that provides robust and stable binding, which is crucial for the proper operation of the FRET mechanism as shown in Fig. 6. Table 4 provides a summary of recent fluorometric aptasensors used for pesticide detection, emphasizing the adaptability and development of FRET-based techniques in the food safety and environmental field.

**Table 4:** Fluorometric aptasensors for pesticide detection

Pesticide	Sample	Linear range	LOD	References
Edifenphos	Water and rice	1.61–19 nM	0.419 nM	[77]
Diazinon	Tap water, tea, and apple	0.33 pM–1.64 µM	76 pM	[78]
Diazinon	Tap water, river water, agricultural runoff water, and urine	4–31 nM	0.4 nM	[79]
Diazinon	13 vegetable and fruit samples	0.33 nM–0.33 mM	22 nM	[80]
Chlorpyrifos		0.29 nM–0.29 mM	2.1 nM	[80]
Malathion		0.3 nM–0.3 mM	2.2 nM	[80]
Malathion	Tap water and matcha	0.01–1 µM	1.42 nM	[81]
Chlorpyrifos	Tap water, vegetable, and fruit samples	5–600 nM	3.8 nM	[82]
Chlorpyrifos	Water samples	0–21.6 nM	0.028 nM	[83]
Acetamiprid	Tap water, wastewater, and tomatoes	1.6–120 nM	0.3 nM	[84]
Acetamiprid	Lake water	0.1–60 µM	0.0559 µM	[85]
Acetamiprid	Chinese cabbage and cucumber	2.3–27 µM	1.9 µM	[71]
Omethoate	NA	4.7 nM-0.94 mM	4.7 nM	[86]
Dimethoate	Tap water and apple juice	1 nM-50 µM	0.218 nM	[87]

### Chemiluminescent Based Aptasensors

Chemiluminescence refers to the light emitted as a consequence of a chemical process, typically including the oxidation of a luminophore, such as luminol, in an environment of hydrogen peroxide (H_2_O_2_). This technique produces light independently, requiring no complex instruments, such as external excitation and fluorescence detectors, hence improving its accessibility [88]. For instance, Qi et al. have applied the luminol-H_2_O_2_ chemiluminescent reaction for the development of a chemiluminescence aptasensor to detect ACE with high sensitivity and selectivity [89]. This research uses the unique integration of aptamers and Au NPs to enhance chemiluminescent signals. The outcome is achieved by the distinctive capacity of aptamers to recognize specific ACE, coupled with the catalytic characteristics of Au NPs. This induces the generation of chemiluminescent signals when H_2_O_2_ and luminol are present. Subsequently, the Au NPs transition from a dispersed state to aggregation as a result of conformational changes in aptamers induced by ACE binding. The changes in the size and shape of Au NPs are detected using their chemiluminescent signal, an innovative approach that emphasizes the sensor’s selectivity. The aptamer’s selectivity guarantees precise detection even in intricate matrices, achieving better sensitivity, with a LOD as low as 62 pM. Similarly, Weng et al. have conducted a fast screening of soybeans for glyphosate [90]. An aptamer specific to glyphosate has been developed for selective binding to glyphosate molecules, while the unbound aptamers are attached to Au NPs. Then the signal exhibited luminol-H_2_O_2_ emission, which is catalyzed by the aggregation of Au NPs in a chemiluminescent process resulting from the glyphosate-aptamer complex. The LOD for this developed aptasensor is 5.3 pM. This aptasensor allows for fast screening of glyphosate residues, which is beneficial for real-time monitoring and quick decision-making in agricultural and food safety fields. The direct and proportional correlation between glyphosate concentration and signal intensity enhances the sensor’s quantitative detection efficiency. These two examples illustrate the ability of the luminol-H_2_O_2_ system in enhancing chemiluminescent signals via the catalytic action of Au NPs. However, the following are the two major challenges in the chemiluminescent aptasensors. Firstly, the sensitivity of the chemiluminescent response is significantly dependent on the precise size and shape of the Au NPs. Variations in synthesis or preparation may impact reproducibility and dependability. Secondly, environmental conditions, including pH and temperature, may influence the chemiluminescent interaction between luminol and H_2_O_2_, which might reduce its effectiveness in field applications.

## Surface-Enhanced Raman Spectroscopy (SERS) Based Aptasensors

**Fig. 7:**
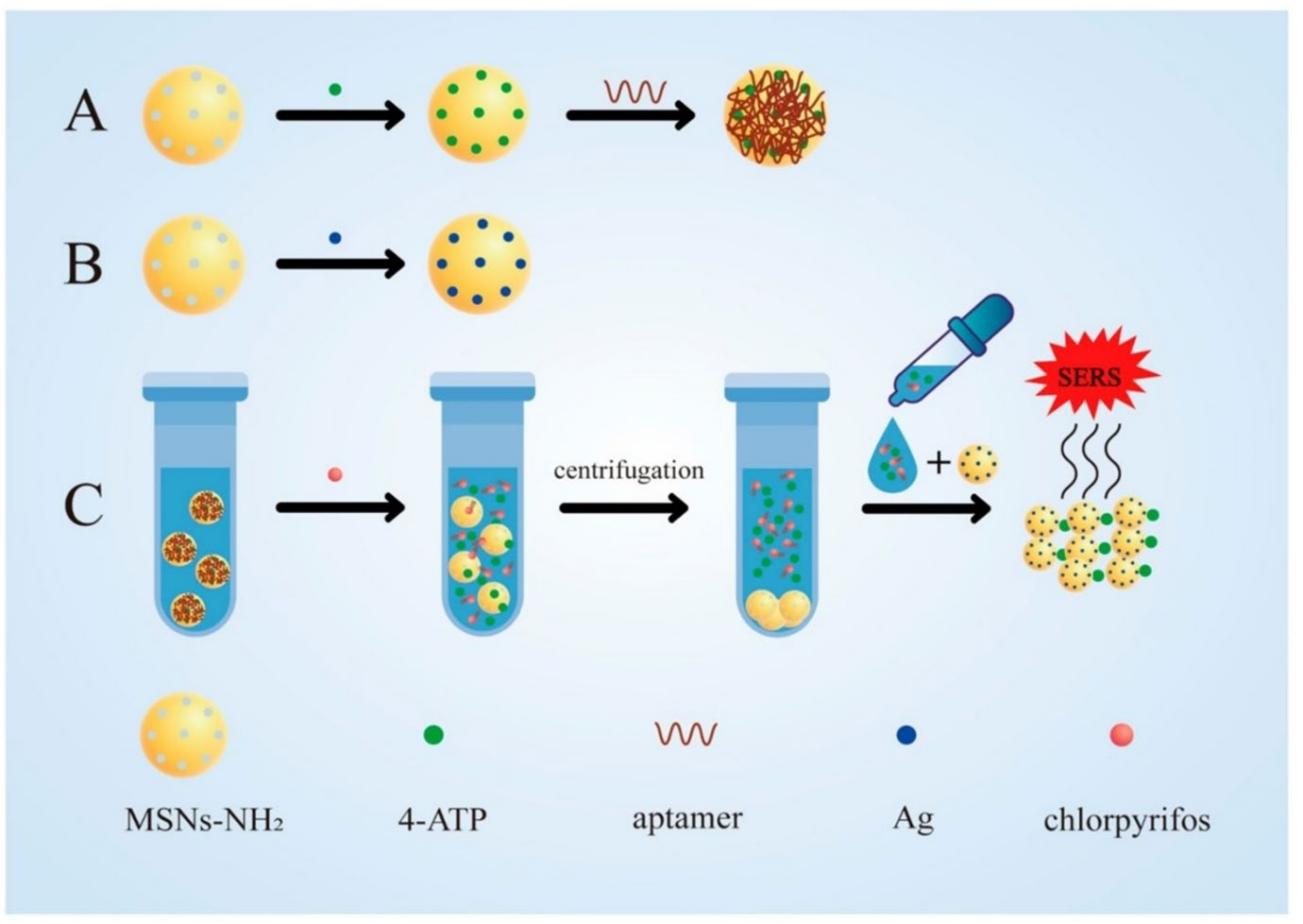
Diagram illustrating a SERS-based aptasensor for the CPF detection. Adapted with permission [92]. Copyright 2022, MDPI

Surface-enhanced Raman spectroscopy (SERS) is a spectroscopic technique that combines Raman spectroscopy with nanotechnology to improve molecular identification and analysis. Utilizing metallic nanostructures, such as Au or Ag, significantly enhances Raman signal intensity. This enhancement, capable of amplifying Raman scattering by up to 10,000 times, arises when target molecules strongly adsorb onto the uneven surfaces of these metals. Compared with FRET, SERS provides some benefits, such as enhanced sensitivity, the capacity to multiplex, a low chance of photobleaching, and less interference from water background [91]. The combination of the specificity and binding affinity of aptamers has garnered attention for the development of aptasensors using SERS to detect pesticide residues with high sensitivity. Recent developments in SERS-based aptasensors have successfully proved these benefits. For instance, Dong et al. have developed a SERS-based aptasensor to detect CPF [92]. As shown in Fig. 7, the signal molecule 4-Aminothiophenol (4-ATP) of SERS is regulated by aptamers, which are incorporated into aminated mesoporous silica NPs (MSNs-NH_2_) using a one-pot approach. This approach for combining signal molecules with an aptamer coating is efficient and simplifies the manufacturing process, which makes them useful to improve the signal stability. The MSNs-NH_2_ are coated with a CPF-specific aptamer by electrostatic interaction. The application of Ag-carrying MSNs as a strengthening substrate significantly amplifies the SERS signal, allowing reliable detection even at minimal analyte concentrations. The aptamer and CPF exhibited specific binding, resulting in the release of the 4-ATP. The quantity of 4-ATP released is directly proportional to the quantity of CPF. The use of aptamers to regulate the release of 4-ATP signaling molecule is novel and offers a direct measurement method of CPF. The proposed aptasensor responded linearly to CPF at concentrations ranging from 71 to 710 nM. The LOD for the developed aptasensor is 57 nM.

## Other Types of Aptasensors

The development of aptasensors that include cutting-edge methods, such as capillary electrophoresis combined with laser-induced fluorescence (CE-LIF), microfluidic chips, and microcantilever technology shows significant progress in pesticide detection. The combination of aptamers with CE [93] and microfluidic chips [94] offers potential pathways toward pesticide detection. By merging the specific binding capability of aptamers with the swift analysis capability of CE, the resulting CE-based aptasensor can significantly enhance the selectivity, reduce detection time, and improve the precision of trace pesticide detection. This concept is further improved by the combination with LIF for the detection of OPs. For example, CE is combined with fluorescent cadmium telluride/cadmium sulfide core/shell QDs that are bound to DNA aptamers and amino-modified oligonucleotides (AMOs) to form the QD-AMO-aptamer complex [93]. Upon target addition, the target selectively binds to the DNA aptamer, resulting in the splitting of the QD-AMO-aptamer complex and the subsequent freeing of the QD-AMO. During the CE-LIF detection procedure, there is a displacement of the peak height ratio within the QD-AMO and QD-AMO-aptamer duplex. Ratiometric analysis enhances reliability by minimizing potential variations, such as changes in QD concentration or photobleaching. QDs serve as effective fluorescent labels for biosensing applications owing to their robust fluorescence characteristics, which include elevated quantum outputs and photostability. When combined the LIF with aptamer-based CE, this design improves accurate detection, improved resolution, and separation efficiency. This approach is used to detect PRO, PHR, Omethoate (OMT), and ISO, achieving LODs of 0.1, 0.2, 0.23, and 0.17 µM, respectively. Despite their improved performance, CE-LIF systems are complicated and may not be readily available for regular usage in situations with limited resources. Portable devices that integrate microfluidics with QD-based biosensing could overcome these restrictions and enhance practicality for on-site detection.

Another technique involves the use of microfluidic chips, which allow the management of small amounts of fluid in microscale channels, enabling the completion of sample preparation, separation, and detection in a single test [95]. Compared with traditional methods, the microfluidic chip provides an integrated and more time-efficient method for determining pesticide residue and can conduct simultaneous multisample analyses [96]. For instance, Fujii et al. have developed an aptamer-based microfluidic chip biosensor for the detection of OMT vapor [97]. An innovative and effective design for vapor absorption and detection combines agarose gel, nanopore technology, and droplet contact techniques to develop a droplet interface bilayer. In this system, the agarose gel efficiently absorbs OMT vapor among the aqueous droplets present inside an oil phase. Its integration with nanopore technology facilitates the signal transmission, demonstrating a synergistic design. The structural modification of the aptamer and the following nanopore obstruction serve as a unique and quantifiable signal for OMT detection. This design enables better sensitivity for detection in solution-phase and vapor-phase, with LODs of 4.8 nM and 469 nM, respectively. Furthermore, the aptasensor’s stability and performance inside the oil phase are maintained by this innovative design and make the biosensor adaptable and versatile for many applications.

Moreover, microcantilever-array aptasensors, which are becoming more compact and scalable, provide a further advanced pesticide detection technology. With the progress in microfluidic chip technology, Li et al. have designed a microcantilever-array sensor based on aptamer incorporation and optical fiber detection [98]. The PRO-specific aptamer is attached to the microcantilever via an Au–S bond. This relationship is shown by the microcantilever’s apparent deflection developed by the particular interaction between the aptamer and PRO. This aptasensor exhibits a linear response to PRO within a concentration range of 13 nM to 2.7 µM. The LOD for this proposed aptasensor is 3.5 nM. The integration of microcantilever technology with optical fibre detection enhances measurement accuracy of deflection and elevates overall sensor performance. The application of microcantilever deflection as a detection signal is an innovative design providing a direct and quantifiable physical reaction to molecular interactions. The integration of microcantilever array and microfluidic chip technology enhances the sensor’s scalability and miniaturization while enabling it to handle small sample quantities. This feature is crucial for the development of portable, point-of-care devices. In addition, the overview of other advanced aptasensors for pesticide detection is shown in Table 5. Additionally, to provide an in-depth analysis of various types of aptasensors, a comparison table has been provided that summarizes important factors, including cost, sensitivity level, advantages and disadvantages, and practicality-based lab scale or field level. Table 6 enables the comparison of the recently developed aptasensors and aids in understanding their advantages and disadvantages in a straightforward manner. The comparative analysis indicates that some aptasensors provide a more favorable balance of cost and performance, while others provide enhanced sensitivity at an increased cost. These results have important implications for aptamer selection and use across a range of fields.

**Table 5:** Other types of aptasensors for pesticide detection

Method	Pesticide	Linear range	LOD	References
Chemiluminescent	ACE	0.8–630 nM	62 pM	[89]
	GLY	5.9 pM–59 µM	5.3 pM	[90]
	ATZ	4.6 pM–0.46 nM	1.53 pM	[99]
	ALD	40 pM–4 nM	9.6 pM	[100]
SERS	CPF	71–710 nM	57 nM	[92]
	CPF	0.29–900 nM	0.19 nM	[101]
	MAL	500 nM–10 µM	500 nM	[102]
	DMT	0.5–10 µM	0.5 µM	[103]
CE	PRO	0.3–10 µM	0.10 µM	[93]
	PHR	0.6–10 µM	0.20 µM	[93]
	OMT	0.7–10 µM	0.23 µM	[93]
	ISO	0.5–10 µM	0.17 µM	[93]
Microfluidic chip	CBF	0.2–50 nM	67 pM	[94]
	OMT	NA	4.8 nM (in solution) and 469 nM (in vapor)	[97]
Microcantilever	PRO	13 nM–2.7 µM	3.5 nM	[98]

*ACE* acetamiprid, *GLY* glyphosate, *ATZ* atrazine, *ALD* aldicarb, *CPF* chlorpyrifos, *MAL* malathion, *DMT* dimethoate, *PRO* profenofos, *PHR* phorate, *OMT* omethoate, *ISO* isocarbophos, *CBF* carbofuran

**Table 6:** Summary of aptasensors characteristics

Aptasensor type	Cost	Advantages	Disadvantages	Practicality
Electrochemical	Moderate	• High sensitivity (LOD at fM to pM level) • Easy miniaturization • User friendly • Fast response • Portability	• Require complex electrode preparation • Susceptible to fouling • Susceptible to changes caused by dissolved oxygen and temperature fluctuations	• Suitable for point-of-care applications and environmental monitoring
Colorimetric	Low	• Simple and inexpensive • Easy interpretation by visual detection • Practicality	• Lower sensitivity (LOD at pM to nM level) • Cannot perform quantitative and multiple detection without the use of other equipment • Prone to aggregation owing to the charged species that exist in the sample	• Ideal for preliminary screening with suitable equipment
Fluorescent	Moderate to high	• High sensitivity (LOD at pM to nM level) • Broad dynamic range • Simple operation • Strong signal-to-noise ratio • Some can be interpreted by visual detection	• Requires florescent labeling • Expensive and complex detection equipment • Fluorescent background and fluorophore’s lifespan impact detection accuracy • Fluorescent probes are sensitive to photobleaching and nonspecific quenchers	• Suitable for lab-based testing and less practical for field use • Ideal for preliminary screening with suitable equipment
Chemiluminescent	Moderate	• High sensitivity • Broad dynamic range • High signal-to-noise ratio • Low background emission • Speed	• Complicate setup • Require multiple reagents and equipment	• Suitable for lab-based testing and less practical for field use
SERS	High	• High sensitivity • Label-free analysis • Multiple-target detection • Non-destructive method	• Expensive and complex equipment • Demands specific substrates • Variability of signals • Needs laboratory environment	• Requires operational skill • Not available as general practice • Suitable for laboratory applications such as molecular analysis etc.,
CE	Moderate to high	• High efficiency of separation and resolution • Minimal sample requirement • Rapid response • Quick analysis • Simplified automation	• Moderate throughput • Restricted to charged substances • Needs high-voltage equipment • Startup costs • Requires operational skill	• Suitable for lab-based testing and less practical for field use
Microfluidic chip	Moderate to high	• Minimal sample and reagent usage • High throughput • Portability	• Requires operational skill • Fabrication difficulty • Startup costs • Possibility of clogging	• Suitable for point-of-care, environmental and lab-on-a-chip applications
Microcantilever	High	• High sensitivity • Label-free analysis • Real time monitoring • Minimal sample requirement	• Complicate setup • Sensitive to environmental factors (humidity, temperature etc.,)	• Suitable for disease diagnostics and environmental monitoring

## Conclusions and Future Perspectives

This review summarizes the recent advances in using aptasensors for the detection of pesticides. Compared with antibody-based and enzyme-based biosensors, aptamer-based biosensors offer advantages in terms of better stability, low cost, and regeneration ability, which make them a compelling choice for various applications. Electrochemical aptasensors are distinguished by their sensitivity, versatility, and capacity for real-time testing. Colorimetric aptasensors that rely on color changes provide a simple and cost-effective detection approach. Fluorescence-based aptasensors stand out for their high sensitivity and rapid analysis. SERS-based aptasensors utilize the enhanced Raman signals for specific and sensitive detection, often with low LOD. The versatility and potential of these aptasensors enable selective analysis of pesticides in various samples, including food and environmental matrices.

Despite significant developments in aptasensors over the last decade, challenges remain. Firstly, appropriate aptamers have not been identified for all pesticides. Establishing systematic and efficient protocols for the creation and selection of high-affinity aptamers for some pesticides remains a considerable challenge. Recent developments in high-throughput sequencing and SELEX have shown promise in the discovery of novel aptamers with great specificity. Furthermore, aptamer selectivity can be improved by adding locked nucleic acids or other modified nucleotides, chemically altering the sequence by truncating or extending it, generating split aptamers or multivalent aptamers, and forming aptamer nanoconjugates. Secondly, most sensors experience nonspecific adsorption at the surface, which affects the accuracy and efficacy of the aptasensor. To mitigate this problem, antifouling coatings, such as polythene glycol or zwitterionic polymers, as well as advanced surface chemistries, such as SAMs or functionalized nanoparticles, have been developed to reduce undesirable nonspecific adsorption. These coatings provide a barrier that inhibits nonspecific binding, therefore enhancing the sensor’s precision in complex matrices, such as environmental and food samples. Thirdly, enhancing the signal-to-noise ratio to guarantee sensitive and reliable detection remains a technical challenge. The integration of nanomaterials, including Au NPs, graphene, carbon, and MOFs, that increase the effective surface area of the sensor has proven to be a successful strategy for enhancing sensitivity. Further investigation into innovative designs for sensing mechanisms and signal amplification systems, such as DNAzyme-assisted aptasensors, dual-mode sensors, or plasmonic enhancement approaches, provides additional possibilities to increase sensitivity. Fourthly, the existence of other compounds in the sample may interfere with the aptasensor’s effectiveness, resulting in false outcomes. Applying strategies, such as shielding, blocking, or using specific coatings and membranes, can reduce interference from other substances in the sample. For instance, silica can be used as a protective and stabilizing layer for electrochemical aptasensor development. Lastly, to mitigate the real-time monitoring difficulties, miniaturizing aptasensors to micro- or nanosized forms would enable on-site detection. Effective modification of aptamers on microelectrodes, such as fiber or wire electrodes, smart systems, and lab-on-chip systems with smartphone integration, also improves the portability and usability in on-site detection. In addition, lateral flow assays provide a straightforward, rapid, and affordable method for on-site detection. Because of their ability to provide visible outcomes in a couple of minutes, these paper-based sensors are suitable for applications that are both quick and user-friendly in a variety of contexts.

With rapid advancements in these areas, these advanced sensing technologies are expected to address environmental and food safety concerns, making them valuable tools for pesticide monitoring and environmental protection in the future.

## Data Availability

No datasets were generated or analyzed during the current study.
